# Cultural adaptation of the Glamorgan Scale to Brazilian Portuguese: Pressure Injury in Pediatrics*

**DOI:** 10.1590/1518-8345.4083.3424

**Published:** 2021-04-12

**Authors:** Marcelli Cristine Vocci, Cassiana Mendes Bertoncello Fontes, Luciana Patricia Fernandes Abbade

**Affiliations:** 1 Universidade Estadual Paulista, Faculdade de Medicina de Botucatu, Botucatu, SP, Brazil.; 2 Scholarship holder at the Coordenação de Aperfeiçoamento de Pessoal de Nível Superior (CAPES), Brazil.

**Keywords:** Translating, Transcultural Studies, Nursing Methodology Research, Pressure Ulcer, Quality of Health Care, Pediatric Nursing, Processo de Tradução, Estudos Transculturais, Pesquisa Metodológica em Enfermagem, Lesão por Pressão, Qualidade da Assistência à Saúde, Enfermagem Pediátrica, Traducción, Estudios Transculturales, Investigación Metodológica en Enfermería, Úlcera por Presión, Calidad de la Atención de Salud, Enfermería Pediátrica

## Abstract

**Objective:**

to describe the methodological process of cultural adaptation of the Glamorgan Scale to Brazilian Portuguese.

**Method:**

a methodological study of translation and cultural adaptation of the Glamorgan Scale, following the six stages: initial translation, synthesis of translations, back-translation, committee of experts, pre-test, and submission of the adapted version to the author for approval. The committee of experts was composed of five physicians and, during evaluation, a semantic, idiomatic, cultural and conceptual analysis was carried out. The agreement and representativeness of the items were assessed using the Content Validity Index. A minimum value of 80% agreement was considered.

**Results:**

all stages of the translation and cross-cultural adaptation process were satisfactory. In the evaluation made by the committee of experts, all items obtained an agreement greater than 80% in the first evaluation round. The pre-test stage allowed for a critical overview of the instrument, where few modifications were suggested by the participants.

**Conclusion:**

the Glamorgan Scale was translated and culturally adapted to Brazilian Portuguese. Future psychometric studies are necessary to validate the scale.

## Abstract (pt)

**Objetivo:**

descrever o processo metodológico de adaptação cultural da *Glamorgan Scale* para o português do Brasil.

**Método:**

estudo metodológico de tradução e adaptação cultural da *Glamorgan Scale*, seguindo as seis etapas: tradução inicial, síntese das traduções, retrotradução, comitê de especialistas, pré-teste e envio da versão adaptada para aprovação ao autor do instrumento original. O comitê de especialistas foi composto por cinco doutores e, durante sua avaliação, foi realizada análise semântica, idiomática, cultural e conceitual. A concordância e a representatividade dos itens foram avaliadas por meio do Índice de Validade de Conteúdo. Considerou-se o valor mínimo de 80% de concordância.

**Resultados:**

todas as etapas do processo de tradução e adaptação transcultural foram satisfatórias. Na avaliação realizada pelo comitê de especialistas, todos os itens obtiveram concordância superior a 80% na primeira rodada de avaliação. A etapa do pré-teste permitiu uma visão crítica geral do instrumento, onde foram sugeridas pequenas modificações pelos participantes.

**Conclusão:**

a Escala de Glamorgan foi traduzida e adaptada culturalmente para o português do Brasil. Estudos psicométricos futuros são necessários para validação da escala.

## Abstract (es)

**Objetivo:**

describir el proceso metodológico de adaptación cultural de la Escala de *Glamorgan* al portugués de Brasil.

**Método:**

estudio metodológico de traducción y adaptación cultural de la Escala de *Glamorgan* siguiendo seis etapas: traducción inicial, síntesis de las traducciones, retrotraducción, comité de especialistas, prueba previa y envío de la versión adaptada para aprobación del autor. El comité de especialistas está formado por cinco médicos y, durante su evaluación, se realizó un análisis semántico, idiomático, cultural y conceptual. Se evaluó la concordancia y la representatividad de los ítems a través del Índice de Validez del Contenido. Se consideró un valor mínimo del 80% de concordancia.

**Resultados:**

todas las etapas del proceso de traducción y adaptación transcultural fueron satisfactorias. En la evaluación realizada por el comité de especialistas, todos los ítems obtuvieron un nivel de concordancia superior al 80 % en la primera etapa de evaluación. La etapa de la prueba previa permitió una visión crítica general del instrumento, en la cual los participantes sugirieron pocas modificaciones.

**Conclusión:**

se tradujo y se adaptó culturalmente la Escala de *Glamorgan* al portugués de Brasil. Se requieren estudios psicométricos futuros para validar la escala.

## Introduction

Pressure Injury (PI) is localized damage to the underlying skin and/or soft tissues, usually on a bony prominence or related to the use of a medical device or other device. It occurs as a result of intense and/or prolonged pressure in combination with shear^(^ 1 ^)^. This condition imposes physical and psychological burdens on the patients and their families and can cause discomfort, pain, impaired quality of life, prolonged hospital stay (a mean of four days^(^ 2 ^)^) and, in severe cases, infected PIs can lead to osteomyelitis^(^ 3 ^)^. In addition, it is related to high mortality rates^(^ 4 ^-^ 5 ^)^, to an increase in the workload of professionals, and to a significant increase in costs for institutions^(^ 6 ^-^ 7 ^)^.

Thus, in the search for improving care, indicators have been studied and, among them, PI stands out as part of the set of quality indicators related to Nursing care processes. In 2010, through the São Paulo Regional Nursing Board, a booklet was launched with the “10 steps for patient safety”^(^ 8 ^)^, one of which is the prevention of PI. In addition, reducing the risk and incidence of PI has become one of the six patient safety goals, and one of the priorities of the Ministry of Health^(^ 9 ^)^.

The prevention of PI requires prior and accurate identification of the risk score of each patient and, related to this, it is essential to implement a set of preventive measures. The availability of reliable instruments that predict the risk of critical pediatric patients developing PI^(^ 10 ^-^ 11 ^)^ is crucial to this process.

For this, the methodological processes^(^ 12 ^)^ of translation and cultural adaptation are essential when the intention is to use an instrument previously developed by researchers from other countries and/or different realities. Instruments for evaluation in the clinical practice have the ability to identify a potential problem, making it possible to concentrate human and material resources to prevent its outcome. In addition, they standardize the conduct to be applied in each situation and organize communication between the health team, which has a direct impact on the priority given to each patient. It is important to emphasize that, behind the use of culturally adapted predictive instruments, there is a vast history of people who have dedicated themselves to making plausible methods that show objectively and quickly the vulnerability of each patient^(^ 13 ^)^.

Pediatric patients, especially in intensive care units, are likely to develop PI^(^ 14 ^)^. Children’s skin is characterized by being immature, thin, sensitive, fragile, poorly protected, and very delicate due to the great immaturity of the structures that constitute it, making it easily breakable^(^ 15 ^-^ 16 ^)^.

In Brazil, the best-known instrument for predicting PI risk for pediatric patients is the Braden Q Scale (B-QS)^(^ 17 ^-^ 18 ^)^, which has good internal consistency (0.93); but its parameters were adapted from an adult version, Braden Scale^(^ 19 ^-^ 20 ^)^, rather than developed specifically for the target population.

The *Glamorgan Scale* (GS), developed in 2009^(^ 21 ^)^, was created from a detailed questionnaire, based on a literature review on PI in the pediatric population, and extensive discussions with pediatric nurses experienced in the prevention and care of PI. From this questionnaire, a survey was carried out with 265 patients admitted to a children’s hospital in England, with the objective of obtaining detailed data on their characteristics. After statistical analysis (Pearson’s chi-square test), variables with statistically significant values (p<0.01) emerged that were used in the composition of the GS.

This scale was designed to specifically assess children and adolescents from birth to 18 years of age, and is also suitable for preterm newborns^(^ 21 ^)^. While other risk assessment scales give similar weights to each subscale, the GS authors showed that some variables, such as mobility, are more significant than others, so they should be weighted according to their impact^(^ 21 ^)^. In a comparative study^(^ 21 ^-^ 22 ^)^, where both scales were applied to 336 pediatric patients, aged between one day of life and 18 years old, the GS showed greater sensitivity (98.4%) and specificity (67.4%) than the B-QS (67% sensitivity and 65% specificity).

When considering the importance of risk assessment for the development of PI in the context of care quality, as well as the better sensitivity and specificity of the GS, this study aimed to describe the methodological process of cultural adaptation of the GS to Brazilian Portuguese.

## Method

This is a methodological study focused on the translation and cross-cultural adaptation of the Glamorgan Scale^(^ 21 ^)^, from English to Portuguese in the Brazilian context.

The GS is composed of nine items that receive scores according to the impairment presented. After assessing each of the nine items, the respective scores are added, obtaining a total score, which varies from 0 to 42 points, where the higher the score value, the greater the impairment and, consequently, the greater the risk of developing PI^(^ 21 ^)^.

According to the GS criteria, there are three risk stratifications for the total score: at risk (10+), high risk (15+), and very high risk (20+). Patients should be evaluated daily, or if their clinical condition changes, or if they are transferred to another unit^(^ 21 ^)^.

**Figure 1:**
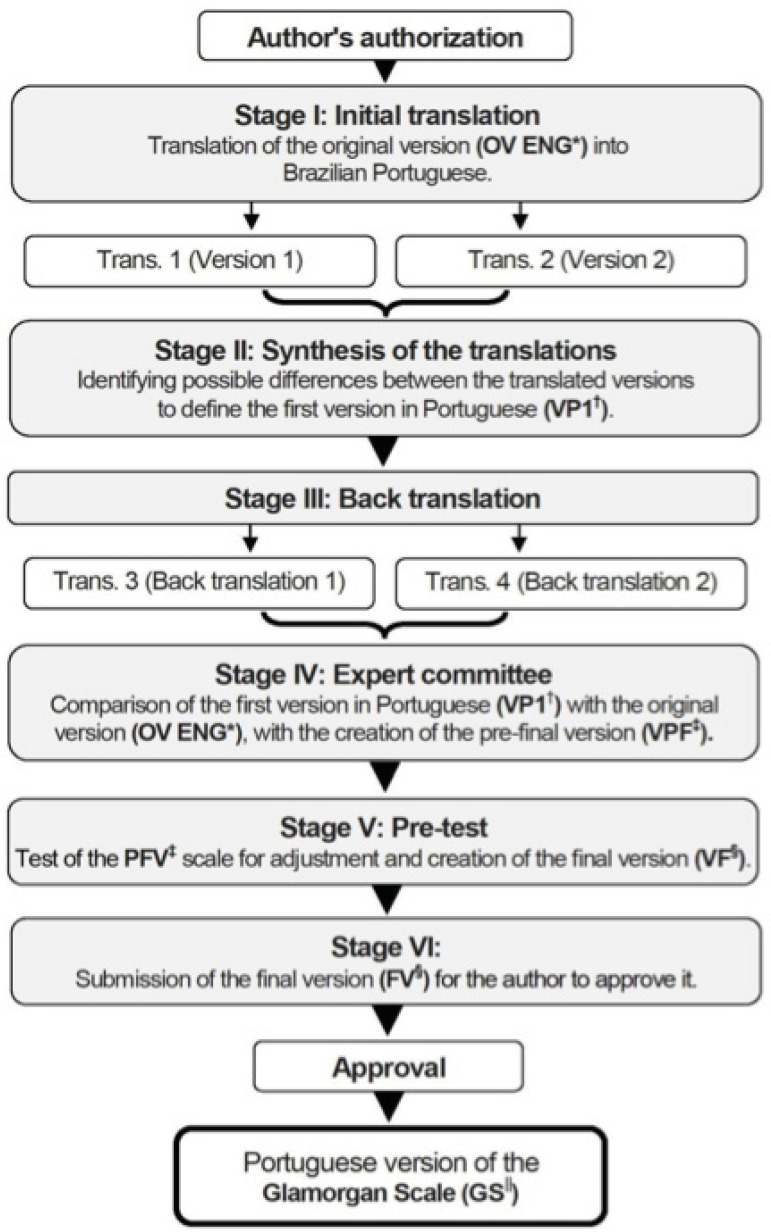
Flowchart of the methodological path of translation and cultural adaptation of the Glamorgan Scale into Portuguese in the Brazilian context. Botucatu, SP, Brazil, 2019 *OV ENG = Original version in English; ^†^VP1 = First version in Portuguese; ^‡^PFV = Pre-final version; ^§^FV = Final version; ^||^GS = Glamorgan Scale

The translation and cross-cultural adaptation process, guided by the theoretical framework of Beaton, et al.^(^ 12 ^)^, comprised the following stages (Figure 1): I: initial translation; II: synthesis of the translations; III: back-translation; IV: committee of experts; V: pre-test; and VI: submission of the adapted version to the author for approval.

Stage I, which corresponds to the initial translation of the original version (OV ENG), was carried out independently by two translators (Trans. 1 and Trans. 2), who were bilingual (English/Portuguese) and had different profiles. Trans. 1, a nurse, had knowledge about the concepts assessed in the instrument, seeking equivalence from a clinical perspective; and Trans. 2 had no knowledge about the health field. After the independent translations, stage II was carried out, in which both translators (Trans. 1 and Trans. 2) established contact for discussion and synthesis of the versions (V1 and V2), resulting in the first Portuguese version (VP1).

In stage III, back-translation, another two translators (Trans. 3 and Trans. 4), who were bilingual (English/Portuguese) and had no training in the health field, did the back-translation of the VP1 instrument, that is, reverse translation to the original language generating two independent back-translations (RT1 and RT2). This is a validity check process, to make sure that the translated version is reflecting the same content as the original version.

In stage IV, the analysis was carried out by the committee of experts. For selection of the committee, it was aimed that the individuals were physicians, fluent in English, health professionals, had vast knowledge in the topic addressed, in addition to having specifically a stomatherapist component specialized in PI, and one had knowledge on the methodological process of cultural adaptation. Based on the translated and synthesized version (VP1), this material was evaluated and compared with the original version. The committee’s main role was to compare the versions, evaluating them as for the semantic, idiomatic, cultural and conceptual equivalencies^(^ 12 ^,^ 23 ^)^. Semantic equivalence allows evaluating the meaning of words in order to preserve their original meaning; idiomatic equivalence evaluates the formulation of expressions and colloquialisms equivalent to the target language; cultural equivalence refers to everyday terms and situations that differ between the cultures; and conceptual equivalence refers to words that have cultural meanings^(^ 12 ^,^ 23 ^)^.

Thus, the experts evaluated and compared the versions, which resulted in the pre-final version (PFV). The relevance and representativeness of the items were assessed using the Content Validity Index (CVI), which measures the agreement among the evaluators. The adequacy of each item varied between adequate and not adequate, and a minimum value of 0.80, or 80%, was considered^(^ 24 ^-^ 25 ^)^.

In the next stage (V), the pre-test was performed, which consisted of the experimental application of the PFV of the scale by professional nurses. Preliminary instruments were delivered to each participant in this phase: the PFV, a script for applying the scale, and the document for recording the evaluation and possible suggestions. Seven intensive care nurses evaluated a group of patients in order to test the scale for understanding, clarity of the questions and answers, and the difficulties encountered by the professionals. The theoretical framework used suggests an ideal of 30 to 40 representatives for this stage^12^. However, the Glamorgan Scale is a clinimetric scale, where the end consumer is the nurse working in the Pediatric Intensive Care Unit. Thus, a convenience sample was selected with the following inclusion criteria: nurses, working in PICUs in high-complexity hospitals, and who were available to participate. Thus, seven nurses were selected, and the researchers premeditatedly waited for them to return their evaluations so as to identify the need to select more participants. Upon receiving the evaluations, compliance was observed, constituting sample saturation and making it unnecessary to expand the sample, since the seven evaluators have equal basic training and high understanding of the text. The suggestions made by the evaluators were accepted and a post-correction version was sent for approval, the final version (FV) thus coming to light.

This version was sent to the authors of the original version (stage VI), obtaining their approval.

In stages I to V, reports were produced by each participating evaluator.

Prior to the research, authorization (personal communication) was granted by the authors who hold the rights to the scale. All the procedures carried out met the ethical principles, and the research was approved by the Research Ethics Committee under Opinion No. 1,908,776.

## Results

In the translation and synthesis stages, the discrepancies found in the translations were related to words or terms with similar meanings in Brazil (e.g., without information and unknown; examining and evaluating; medical record and record). Thus, all the divergences found were studied and the translators, together with the researcher, chose the term they considered most usual in the context.

In the back-translation, versions RT1 and RT2 were identical in 13 statements (65%), and the differences found were evaluated as being synonymous words. Thus, it was concluded that the back-translation versions corresponded to the original instrument.

The OV ENG and VP1 versions were sent to the committee of experts, so that they could make a comparison between them regarding the semantic, idiomatic, cultural and conceptual equivalences. The items’ percentage of agreement was calculated based on the CVI, where all items obtained an agreement greater than 80% in the first evaluation round. In addition to the experts’ assessment of whether the terms were adequate or not, they presented a report with suggestions for changes and their justifications. The experts suggested 16 words that should undergo changes in terms of textual equivalences, including: 12 semantic; 3 cultural; and 1 conceptual. After the experts’ consensus, the suggestions were analyzed by the researchers, where all the recommendations regarding the textual content were deferred to create the pre-final version (PFV), with subsequent application in the pre-test.

After the stage of analysis by the specialists, the pre-test was carried out, which included seven intensive care nurses. At this stage, doubts arose regarding the “mobility” set which, in its composition, has four sub-items, only one of which must be scored. In a report, the evaluators pointed out this issue as a potential confounder, and suggested the identification of the sub-items as being components of the large “mobility” set. In addition to this issue, it was also suggested to list the items for better viewing.

One of the evaluators, in the impaired peripheral perfusion item, highlighted the term “livedo” which, although appropriate, it is little known in the practice, which can hinder its interpretation. Thus, substitution was suggested, or the addition of the term mottled or mottling.

**Figure 2:**
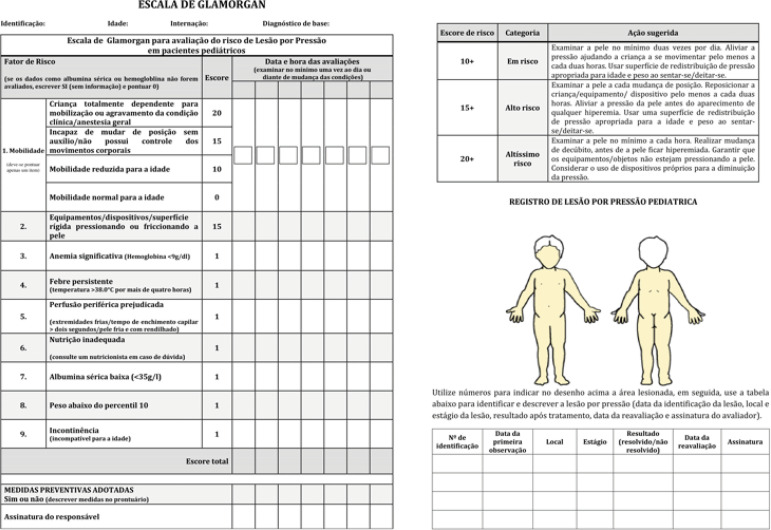
Brazilian version of the Glamorgan Scale after the methodological process of translation and cultural adaptation. Botucatu, SP, Brazil, 2019

After reviewing these items, the final version (FV) was attained, which was sent to the authors of the original version for approval. They approved the FV of the scale, and the Brazilian version of the Glamorgan Scale was created, as shown below (Figure 2).

## Discussion

The Nursing team plays a fundamental role in the early identification and implementation of strategies for the prevention of PI, highlighting the role of nurses, who seek new knowledge to support their practice. However, the prevention of PI is still a challenge for Nursing.

A study carried out in a public hospital in Brazil applying the B-QS in a PICU pointed out a high risk for the development of PI in 87% of the patients and development of 24 PIs^(^ 14 ^)^, thus highlighting the importance of implementing preventive protocols^(^ 26 ^)^ and predictive instruments for the effective prevention of this condition. Another study confirmed that all patients who developed PIs had in fact a high risk alert when applying the scale^(^ 27 ^)^.

In view of the proven high risk, it is necessary for the professionals to know about the predictive risk instruments. Currently, the B-QS is the most used and most reported instrument in the scientific literature for pediatric patients admitted to critical care^(^ 11 ^,^ 28 ^)^. As previously stated, the B-QS is an effective instrument; however, the GS was specifically developed for the study population, having greater accuracy, greater predictive capacity, and can even be applied to a broadest age group (0 to 18 years old)^(^ 29 ^)^. For this reason, the cross-cultural adaptation of this new instrument was carried out.

The translation and cross-cultural adaptation of instruments are important processes to ensure the accuracy and reliability of the measurements obtained by health professionals and researchers. Once created in a particular country, an instrument can be used in different countries. For this proposal, an adequate translation and cross-cultural adaptation must be carried out, taking into account the characteristics of the context in which it will be inserted. The process of cultural adaptation of instruments is a legitimate procedure, capable of promoting the exchange of knowledge among researchers, being also a financially accessible and important method for comparing the results of studies using the same tool^(^ 30 ^-^ 31 ^)^.

There are numerous strategies for the process of cross-cultural adaptation, ranging from simple translation by researchers to the most detailed process^(^ 12 ^)^. During the process, all the stages of this study sought to adjust the instrument to the target population, and efforts were directed so that this tool could be applied in all Brazilian hospitals.

The analysis by the committee of experts regarding the textual equivalences was essential to ensure that the new instrument is understandable by nurses. The experts made significant suggestions, making the instrument clearer and culturally appropriate for Brazilian Portuguese. Evidence shows that research studies evaluated by a committee of experts have better indexes of adjustments of the models, with more adequate content for the proposed context^(^ 32 ^-^ 33 ^)^. All the suggestions regarding the textual content were accepted for the creation of the pre-final version (PFV).

The application of the pre-test allowed for a critical overview of the instrument, where doubts arose on how to score the “mobility” item. Following the participants’ suggestions, the word “mobility” was added in front of its subcategories, and below the phrase “only one item must be scored”.

Therefore, it was not necessary to change the number of items that make up the scale or the way it is evaluated; thus, the Brazilian version of the Glamorgan Scale is also composed of nine items, with a total score from 0 to 42.

The translation of an instrument in a region of Brazil, a country with a homogeneous language, can be considered appropriate for the entire national territory. It is also believed that the new version of the instrument can be used by professionals from other countries whose native language is Portuguese.

Considering that the GS was specifically designed for children and adolescents from birth to 18 years of age, and that it has greater specificity and sensitivity than the B-QS, this study contributes to the field of health and nursing by bringing a specific tool to assist the professional nurse in the risk assessment of PIs in the pediatric population, allowing specific preventive interventions to be incorporated in the assistance provided to high-risk patients.

As a limitation, it should be noted that the scale has not yet undergone the process of cultural adaptation in another country, making it difficult to discuss and compare the results.

## Conclusion

This study allowed for the translation and cross-cultural adaptation of the Glamorgan Scale in the Brazilian Portuguese version, contributing to the advancement of knowledge and evidence-based practice, insofar as, in a relevant manner, it provides an instrument capable of evaluating the risk of critical pediatric patients developing PIs.
